# Remote cognitive assessment to early detection of cognitive decline

**DOI:** 10.18632/aging.204908

**Published:** 2023-07-14

**Authors:** Gabriele Cattaneo, Javier Solana-Sanchez, Josep Maria Tormos-Muñoz

**Affiliations:** 1 Institut Guttmann, Institut Universitari de Neurorehabilitació adscrit a la UAB, Spain; 2 Universitat Autònoma de Barcelona, Spain; 3 Fundació Institut d’Investigació en Ciències de la Salut Germans Trias i Pujol, Spain

**Keywords:** cognitive assessment, computerized cognitive assessment, memory, aging, cognitive decline, mHealth

Thanks to advances in medicine in the last decades, human life expectancy has increased considerably with an enormous impact in worldwide demographic distribution [1]. In the developed world the number of people older than 65 years surpassed the number of children younger than 15 years, and the World Health Organization (WHO) forecasts that for 2050 the number of older adults will double those of young people.

Even though this could be apparently encouraging, it has serious implication for public health, considering that advancing age is the main risk factor for the development of major neurological diseases, which are the first cause of disability worldwide [2].

Indeed, one in four people in the world suffers brain-related disability nowadays [2], and according to WHO, by 2030 half of the worldwide economic impact of disability will be due to brain-related disability. This is a staggering global challenge, and its magnitude continues to grow, affecting not only the patient, but also their families, friends, and society at large.

In this context, age-related cognitive decline is one of the leading public health challenges to deal with [3] and an increasing number of studies and initiatives are trying to find effective strategies to early detection of cognitive changes to implement effective intervention that could reduce the impact of developing cognitive impairment, Alzheimer’s disease, or any other kind of dementia [4]. Moreover, once detected and diagnosed, these conditions should be accompanied by an appropriate and efficient follow-up, to monitor their evolution and evaluate the effects of prescribed interventions.

In this sense, self-administered computerized cognitive assessment, given its low cost and high scalability, could represent a useful and valuable solution. Indeed, it would allow to run periodic and repeated population large-scale screenings from middle to old age adults and detect early and subtle cognitive changes, indicating the presence of cognitive decline, allowing earlier interventions that could revert or attenuate its progression. Alternatively, in the presence of a clinical diagnosis, these digital solutions would have a great impact in terms of sustainability, facilitating the access to clinical services, by allowing repeated and periodic cognitive testing to monitor patient’s evolutions over time and guiding the therapeutic plan on a more efficient manner.

Recently we presented “Gutmann Cognitest”^®^, a digital solution for self-administered computerized cognitive assessment. It has been initially validated in a sample of middle age adults participating in the Barcelona Brain Health Initiative (BBHI) [5], showing a good usability and satisfactory reliability [6].

In terms of component structure and convergent validity, this digital solution was shown to be able to satisfactorily measure main cognitive domains and functions like memory, executive functions, visuo-motor speed, and mental rotation. Divergent validity showed some degrees of overlap, in line with what typically can be found in healthy and clinical populations for classical paper and pencil neuropsychological test [7]. This overlap may suggest that a global cognitive score, also validated in the paper, can be useful to inform about general cognitive functioning, and could be used as the more suitable index for populational screening and monitoring patients over time. This global cognitive score is also in line with the proposal from Wilson and collaborators [8], who decades ago already stated that a global cognitive functioning index is a single robust marker of functional decline over time. In an ageing world, digital solutions like “Guttmann Cognitest”^®^ will definitely play a key role in helping to provide valuable clinical services, allowing efficient monitoring of cognitive functioning over time.
